# No relationship between gender stereotypes and mental rotation in preschool girls

**DOI:** 10.3389/fpsyg.2025.1650979

**Published:** 2025-09-16

**Authors:** W. Miro Ebert, Leonardo Jost, Petra Jansen

**Affiliations:** Faculty of Human Sciences, University of Regensburg, Regensburg, Germany

**Keywords:** spatial ability, gender stereotypes, human sex differences, preschool, children, mental rotation, kindergarten

## Abstract

Gender stereotypes about spatial ability have been proposed as a contributing factor to the gender gap in STEM. This goal of this study was to investigate whether implicit gender stereotypes regarding spatial ability are associated with mental rotation (MR) performance in preschool-aged girls, and whether visuospatial working memory (VSWM) plays a moderating role. Fifty-two girls aged 5 to 6.75 years completed a chronometric MR task, a computerized Corsi block-tapping task (assessing VSWM), and a single-target Implicit Association Test measuring associations between gender and toys used in spatial play. Participants did not show significant implicit stereotypes favoring either gender. Contrary to our hypotheses, no evidence for a relationship between implicit stereotypes and MR response times was found. Unexpectedly, stronger implicit associations linking boys with spatial ability were associated with higher MR accuracy. VSWM was positively correlated with both MR accuracy and implicit stereotype scores. Since the hypothesized relationship between implicit stereotypes and MR was not observed, a potential moderation through VSWM was not examined. Our findings diverge from prior research suggesting that stereotype-consistent associations in girls are linked to lower spatial task performance. Taken together, the results cast doubt on the robustness of implicit gender stereotypes about spatial ability in early childhood and highlight the complexity of their potential impact on spatial cognition. Further research is needed to clarify under what conditions, if any, such stereotypes affect performance in young children.

## 1 Introduction

Across the lifespan, spatial ability and the skills that comprise it—such as spatial visualization, spatial orientation, perspective taking and mental rotation (MR)—are useful in a wide range of contexts. In childhood, spatial skills are required to engage in activities such as games, puzzles, and sports. As adults, we rely on spatial skills to, for example, navigate our environment, or give directions (Newcombe, 2020). Aside from their relevance to daily life, spatial skills are crucial in specific professional and academic fields. Especially in science, technology, mathematics, and engineering (STEM; Newcombe, 2017), spatial ability has long been considered critical (see, e.g., Super and Bachrach, 1957). Research shows that spatial ability can predict the choice to pursue a career or education in STEM (Wai et al., 2009) and success in these endeavors (Newcombe and Shipley, 2015).

For over two decades, the underrepresentation of women in STEM fields has been a topic of political and academic interest (see, e.g., Hanson, 1996; Wang and Degol, 2013). Although there are around 3 million more women in STEM now than there were in 2011 in the United States, their representation remains low at around 34% of the country’s total STEM workforce (National Science Board, 2024). In Europe, women comprised 41.3% of the employed scientists and engineers in 2019 (European Commission, Directorate-General for Research and Innovation, 2021). The authors suggest that “given the strategic importance of technology (tech) industry to the EU economy, these data indicate that greater effort is needed to increase women’s participation in this field” (European Commission, Directorate-General for Research and Innovation, 2021, p.60). This claim is further underscored by the underrepresentation of women among doctoral graduates in most STEM fields (European Commission, Directorate-General for Research and Innovation, 2021).

Researchers have proposed a variety of explanations for the disparity in STEM (see Christie et al., 2017; Ertl et al., 2017). Gender stereotypes, for instance, have garnered significant attention. Nosek et al. (2009) outline that STEM subjects and professions are often perceived as male domains, which may contribute to sex differences in STEM participation and achievement. Matching this rationale, the authors demonstrated, based on data from 34 countries, that national implicit gender-science stereotyping related to sex differences in eighth-grade math at a national level (Nosek et al., 2009). Moreover, cognitive factors—particularly spatial ability—are often introduced as a possible driver for the gender disparity in STEM. As Levine et al. (2016) discuss, sex differences have been reported on various spatial tasks and are especially pronounced in MR (e.g., Voyer et al., 1995), the cognitive process of imagining an object turning in space to determine its orientation or match it to another object. Mixed findings from infant and child studies (see Levine et al., 2016) leave open questions about when sex differences in spatial ability emerge, how they develop, and what underlying factors contribute to them. As with sex differences in STEM, gender stereotypes have been proposed as a mechanism underlying the sex differences in MR. Research suggests that spatial ability, which is deemed crucial in many STEM contexts, is commonly viewed as a male quality by children (e.g., Vander Heyden et al., 2016; Ebert et al., 2024a) and adults (e.g., van der Ham and Koutzmpi, 2023). Furthermore, numerous studies demonstrate that stereotype related effects can play a role in spatial task performance. Especially concerning MR, research is abundant demonstrating stereotype threat and lift effects in adults and adolescents (e.g., McGlone and Aronson, 2006; Moè and Pazzaglia, 2006). Other studies show an influence of the stereotyped nature of MR stimuli on task performance of adults (Rahe et al., 2021; Rahe and Jansen, 2022). Apart from these effects, a study in university students suggests that unconscious stereotypic beliefs, commonly referred to as implicit stereotypes, correlate with task performance (Sanchis-Segura et al., 2018). Moreover, stereotype endorsement appears to be critical to stereotype threat effects, according to a study in high-school students (Moè and Pazzaglia, 2006). Considering such findings, the relevance of stereotypes in the development of spatial ability, and by consequence, the development of sex differences in STEM, constitutes an interesting research field. A recent study suggests that five- and six-year-old children hold stereotypic beliefs about spatial ability on an implicit and explicit level (Ebert et al., 2024a). No clear relationship between stereotyping and MR performance was found in this study. However, in girls, but not in boys, implicit stereotypes were related to MR accuracy. Girls who implicitly associated boys with spatial ability more strongly solved the task less accurately (Ebert et al., 2024a).

Another factor that has been implicated in spatial ability in general and MR specifically is working memory—the cognitive system responsible for the temporary maintenance and manipulation of information. MR is believed to involve multiple stages of processing (Shepard and Cooper, 1982; Just and Carpenter, 1985; Heil and Rolke, 2002), some of which entail keeping an object representation in mind and manipulating it (i.e., identification, discrimination, and mental rotation phases). Given these demands, it comes as no surprise that working memory is thought to be centrally involved in MR. Especially the visuospatial sketchpad, which is concerned with information about object features and location (Baddeley and Hitch, 1974), has been the subject of several studies on MR. In fact, studies in children (Lehmann et al., 2014) and adults (e.g., Kaufman, 2007) implicate visuospatial working memory (VSWM) and its subcomponents in MR (Hyun and Luck, 2007; Ebert et al., 2024b).

Crucially, evidence from studies with adults and adolescents also suggests links between stereotype effects and working memory. A prominently proposed mechanism underlying stereotype threat effects concerns disruptions in working memory capacity (Schmader, 2010). The fear of being evaluated negatively and/or confirming a stereotype about one’s group is assumed to occupy part of an individual’s working memory capacity, thereby affecting performance in tasks that rely on this resource. A study by Schmader and Johns (2003) provided evidence that the effect of stereotype threat on women’s math performance was completely mediated by working memory capacity. A study in first-year psychology students by Bonnot and Croizet (2007) suggests that stereotype internalization can affect performance through the same mechanism (i.e., interference in working memory). Adding to these findings, Régner et al. (2010) found working memory capacity to moderate stereotype threat effects in a sample of university students. Only individuals with low working memory capacity were susceptible to stereotype threat induction in their study. Similar results come from a study in adolescents from Hong Kong (Chan and Rosenthal, 2014). These studies used complex span tasks to assess general working memory capacity and mostly focused on mathematical performance as the outcome variable. To the best of our knowledge, this topic has not been researched in a younger population. Regarding studies that include VSWM specifically, a study in adult athletes found moderating effects of VSWM capacity on stereotype threat effects on a simple motor task (Laurin et al., 2022). Thus, while working memory capacity is usually assessed more generally in examining its relation to stereotype effects, the visuo-spatial subsystem has been implicated in such processes empirically. Given the role of VSWM in MR, we believe investigating whether VSWM capacity relates to stereotype effects in MR is worthwhile.

As outlined above, MR shares relationships with stereotypes and working memory. Moreover, evidence suggests that specific stereotype effects may be related with interference in working memory. Therefore, we examine whether implicit stereotyping affects MR in kindergarten girls. If our findings support this notion, we will further investigate whether such an effect would be moderated by working memory capacity. Since, theoretically, VSWM would be expected to affect performance through its relevance to the later perceptual stages (identification and discrimination) and the rotation process, we want to examine both general effects on MR performance and effects emerging in the rotation phase. The corresponding hypotheses are the following.

*H1*: Stronger implicit beliefs linking spatial ability to males, will predict lower MR performance in girls.

*H1.1*: Stronger stereotypic beliefs will predict a less efficient rotation process, indicated by steeper increases in response time and/or decreases in accuracy with increasing rotation angle.

*H2*: The relationship between implicit stereotypes and MR in girls will be moderated by working memory capacity.

*H2.1*: The relationship between implicit stereotypes and efficiency in the rotation process in girls will be moderated by working memory capacity.

These expectations generally build on the assumption that negative stereotypes about one’s group impair performance through disruptions in working memory. Consequently, the same effects could be expected in boys holding negative stereotypes about the spatial ability of their own gender. However, few boys tend to demonstrate such stereotypes, which is why we focus on girls in this study.

## 2 Method

### 2.1 Participants

A power analysis conducted with G*Power (Faul et al., 2007) indicated that 53 female participants were needed to achieve a power of 0.80 for detecting small-to-medium effects ( $f^{2}$ = 0.12) at an alpha level of 0.05. Data were gathered as part of an intervention study carried out in the city and district of Regensburg, Germany, with all participants recruited via local kindergartens. Among the children who participated, 54 were female, and their pre-test data were used to examine the stated hypotheses. Two participants had to be excluded, due to missing data. Hence, our final sample consisted of 52 kindergarten girls (mean age: 5.88 years, SD = 0.45, range: 5 to 6.75 years). To acknowledge their participation, children received small gifts, while kindergartens received financial compensation. This study was preregistered at https://osf.io/32nc9, and ethical approval was granted by the ethics board of the University Clinic of Regensburg (protocol number: 23–3551-101).

### 2.2 Materials

The study was run on a Lenovo Thinkpad laptop computer with an external 14-inch touchscreen monitor (1920 x 1080px) placed approximately 45 cm in front of the participants. Children used the touchscreen to solve the working memory task. To implement the MR task and the implicit stereotype measure, we used the opensource software OpenSesame (Mathôt et al., 2012), children used large, colored buttons to respond to stimuli in these tasks.

#### 2.2.1 Visuospatial working memory task

**Figure 1:**
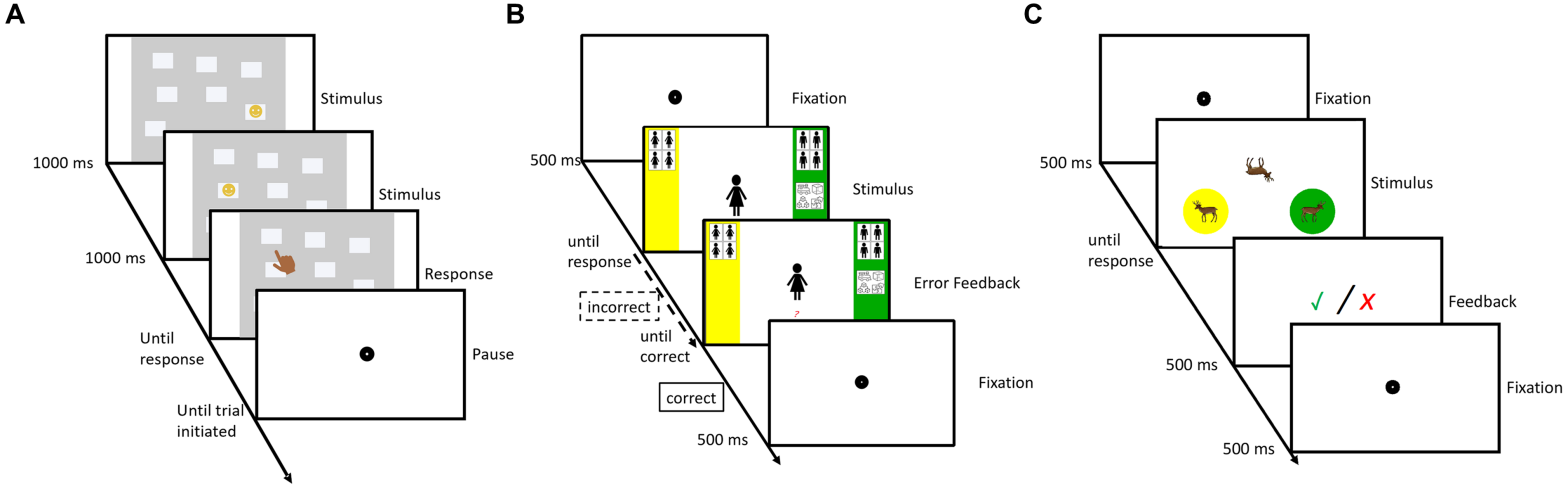
Schematic representations of trial sequences. The leftmost **(A)** schematic displays an exemplary trial sequence of the Corsi block-tapping test. For parsimony, the stimulus presentation (950 ms) and the interstimulus interval of 50 ms are presented together as stimulus. An example of a implicit association test trial is shown in the middle **(B)**. Error feedback was not displayed if the initial answer was correct. The panel on the right **(C)** provides an example of a trial of the mental rotation task.

To measure VSWM, we used a computerized Corsi block-tapping test (Hasselhorn et al., 2012). The task involved an array of nine unsystematically arranged grey fields that remained visible throughout. Each trial presented a sequence of at least two positions, indicated by the appearance of a smiley face for 950 milliseconds (ms) in the respective fields. There was a 50 ms interstimulus interval. Immediately after each sequence, participants reconstructed the order by tapping the corresponding fields on touchscreen monitor (see Figure 1A). They had to remember both the positions and the order they appeared in. Task difficulty adapted dynamically: the sequence length increased when participants responded correctly (sometimes requiring several consecutive correct trials) and decreased after multiple consecutive errors. The task included ten critical trials. The longest correctly solved series among these trials, the Corsi span, was used to quantify VSWM. Before the main task, participants completed two or three practice trials, depending on whether the first trial was solved correctly.

#### 2.2.2 Implicit stereotypes measures

We used an adapted single target implicit association test (ST-IAT; Karpinski and Steinman, 2006) to measure implicit associations between gender and toys commonly used in spatial play (Lego, building blocks, puzzle, toy train). Gender was represented by images of faces of boys and girls. Toys used in spatial play were also presented as images. Children completed five blocks (three practice blocks, two critical blocks), for a total of 144 trials. In the first practice block only faces were presented over 16 trials. The second practice block (16 trials) included both faces and toys. In this phase, toys shared a response button with the faces of one gender—for example, boys and toys were assigned the left response button, while girls were assigned the right response button. This practice phase was followed by a critical block of 48 trials, using the same response assignment. Next, in a third practice block of 16 trials, the response assignment for the toys was switched. If boys and toys had previously shared a response button, girls and toys would now share a response button instead. This response assignment was retained for the second critical block of 48 trials. When participants gave an incorrect response, a red question mark appeared beneath the stimulus, signaling the need for correction before proceeding with the task. Response times were recorded at the time that the correct answer was given (Cvencek et al., 2011). A fixation dot was presented for 500 ms in between trials (see Figure 1B for schematic representation). Response assignments and block order were counterbalanced. ST-IAT scores, were calculated by subtracting the mean response time in the *boys + toys* block from that in the *girls + toys* block (see, e.g., Bluemke and Friese, 2008). Positive scores indicated a stereotype favoring boys.

#### 2.2.3 Mental rotation task

Participants completed a chronometric animal MR task. The stimuli were colored drawings of 12 different animals (camel, crocodile, dog, donkey, elephant, grizzly, lion, pig, rhino, sheep, turtle, and zebra) taken from Rossion and Pourtois (2004). In each trial, three drawings of the same animal were presented simultaneously: one in the center of the screen and two reference images at the bottom left and right. The reference images were always upright, facing outward, with the left image in a yellow circle and the right in a green circle. The central image appeared either upright or rotated by 90°, 180°, or 270° and could always be identified as facing left or right. The upright position served as a reference for determining its facing direction. In half the trials it faced left, in the other half, it faced right. Participants had to determine whether the centrally displayed animal matched the left or right reference image in orientation, mentally rotating it if necessary. They pressed the yellow button if it matched the left image and the green button if it matched the right. Before starting the computerized task, children completed five practice trials using printed images and manipulable cut-outs. This was followed by 16 computerized practice trials. The main experiment consisted of 72 critical trials (12 animals × 2 orientations × 3 rotation angles), with one rotation angle omitted per animal per orientation to shorten the task compared to previous versions (Ebert et al., 2024a). Each stimulus set remained on screen until a response was given. Feedback was provided after every trial: a green checkmark appeared for 500 ms for correct responses, while a red “x” was shown for incorrect responses. Before the next trial, a fixation dot was displayed for 500 ms (see Figure 1C). Participants could take a break after every eight trials, with a mandatory break after 36 trials. Response time and accuracy were recorded. Similar tasks have been used in previous research with kindergarten children (Hahn et al., 2010; Ebert et al., 2024a).

### 2.3 Procedure

Three experimenters conducted the study, with at least one of them present during each data collection session. Testing was conducted individually in a quiet room at the participant’s kindergarten. Consent forms, signed by the participants’ parents, were handed to the experimenter by kindergarten staff. Before participation, the general procedure was explained to each child, and they were asked if they wanted to participate. All participants completed the tasks in the same order. First, they were introduced to the Corsi Block Task as a game in which they had to memorize routes. They first completed practice trials, and then the main task. Following a short break, they were introduced to the ST-IAT, described as a picture-sorting game. Each block was preceded by specific instructions outlining the response mapping for that block. In between blocks, children were encouraged to take breaks. After another short break, they completed the Animal MR Task. This task was framed as a game requiring them to imagine how animals would look when rotated. To introduce the concept, five practice trials involved manually rotating cut-out figures before moving on to computerized trials.

### 2.4 Statistical analyses

Per participant, MR response times more than two standard deviations above or below their individual respective mean response time, per rotation angle, were excluded from response time analyses. This applied to 209 observations in our sample. MR response times faster than 300 ms were excluded from response time and accuracy analyses. There were 17 such responses in our data. In addition to the preregistered plan, response time outliers were excluded based on the 1.5*IQR rule since extreme values were present in the data after outlier exclusion. We excluded 201 observations from response time analysis based on this criterion.

To test whether children held implicit gender stereotypes regarding spatial ability, we conducted a two-sided one sample t-test. To examine hypotheses 1 and 1.1 we fit generalized linear mixed models (GLMMs) to our data using the lme4 package (version 1.1.37; Bates et al., 2015) in R (version 4.5.0; R Core Team, 2025). MR accuracy was modeled using a binomial GLMM as recommended by Dixon, 2008. MR response time was modeled in an inverse gaussian GLMM. We included the nested random factors participant and kindergarten in all models before model reduction. Based on research by Barr et al. (2013), Bates et al. (2015), and Matuschek et al. (2017) we reduced the random effects structures of our respective maximal models following a backward selection approach [likelihood ratio tests (LRTs with *α*-level of 0.2)]. The independent variables were scaled based on their respective standard deviations to facilitate model convergence. Since there were indications of a ceiling effect in MR accuracy (see Supplementary Figure S1), we examined model diagnostics using plots and the DHARMa package (version 0.4.7; Hartig, 2024).

## 3 Results

Descriptive statistics and correlations among the measured variables are shown in Table 1. There was a significant correlation between MR accuracy and Corsi span (*r* = 0.495, *p* < 0.001). The Corsi span also significantly correlated with IAT scores (*r* = 0.314, *p* = 0.023). No other correlations reached significance.

**Table 1:** Descriptive statistics and correlations among study variables.

Variable	Mean	SD	Correlations (df)		
			2	3	4
1. IAT	−36.54	239.90	0.221(50)	0.190 (50)	0.314 (50)*
2. MR Accuracy	0.88	0.13	–	0.168 (50)	0.495 (50)***
3. MR response time	1761.15	343.78		–	0.115 (50)
4. Corsi span	3.90	0.69			–

*p < 0.05; ***p < 0.001.

The girls in our sample did not exhibit implicit stereotypes *t* (51) = −1.098, *p* = 0.28, *d* = 0.15 favoring boys or girls. Table 2 shows model coefficients and test statistics of the inverse gaussian GLMM of MR response times. There was a significant main effect of rotation angle on MR response times (*χ*^2^(1) = 58.53, *p* < 0.001). Greater rotation angles were associated with longer response times. No other effect reached significance in this model.

**Table 2:** Generalized linear mixed model for the dependent variable MR response time.

Predictor	Estimate	95% CI		Test statistic	*p*-value
Intercept	2412.5	1241.87	3417.89		
IAT	2594.9	−4225.62	6492.42	*χ*^2^(1) = 0.58	0.447
Angle	546.8	497.10	633.15	*χ*^2^(1) = 58.53	<0.001
Age	−913.0	−2015.01	122.51	*χ*^2^(1) = 1.89	0.170
IAT * Angle	4156.2	1498.64	10896.73	*χ*^2^(1) = 1.90	0.168

This model included random intercepts by institution and participant, and random slopes for rotation angle by participant. Confidence intervals are based on bootstrapping of linear mixed model with 1,000 simulations.

Table 3 contains model coefficients alongside test statistics of the binomial GLMM of MR accuracy. There were significant main effects of angle (*χ*^2^(1) = 19.57, *p* < 0.001) and IAT score (*χ*^2^(1) = 4.46, *p* = 0.035) in this model. Greater rotation angles were associated with lower accuracy and greater IAT scores were linked to higher accuracy. Since the overall proportion of correct responses was relatively high at 88%, we ran diagnostics to evaluate potential model misspecification. Model diagnostics using the DHARMa package (Hartig, 2024) showed no evidence of overdispersion (*p* = 0.88), outliers (*p* = 0.85), or non-uniform residuals (*p* = 0.14). Visual inspection of the Pearson residuals against fitted values revealed a slight fan-shaped pattern and some extreme residuals at high predicted values, consistent with a potential ceiling effect. However, these are typical for binomial models with high probabilities and were not accompanied by signs of poor model fit.

**Table 3:** Generalized linear mixed model for the dependent variable MR accuracy.

Predictor	Estimate	95% CI		Test statistic	*p*-value
Intercept	4.67	−1.03	9.93		
IAT	32.99	1.05	62.60	*χ*^2^(1) = 4.46	0.035
Angle	−1.20	−1.64	−0.74	*χ*^2^(1) = 19.57	<0.001
Age	−0.72	−5.96	4.97	*χ^2^*(1) = 0.05	0.832
IAT * Angle	−13.27	−44.31	18.73	*χ^2^*(1) = 0.85	0.358

This model included random intercepts by institution and participant, and random slopes for rotation angle by participant. Confidence intervals are based on bootstrapping with 1,000 simulations.

## 4 Discussion

The current study set out to examine potential relationships between implicit stereotypes regarding spatial ability, VSWM and spatial task performance in preschool-aged kindergarten girls. We assessed VSWM, implicit stereotypes and MR performance of participating children.

### 4.1 Gender stereotypes

We found no evidence of implicit stereotypes in our sample. In a previous study using the same IAT (Ebert et al., 2024a), implicit stereotypes were observed in a mixed-gender sample, though the effect was small (*d* = 0.17). Notably, when results were broken down by gender in that study, no significant effects emerged (both gender-specific *ds* = 0.17). In the current study, we observed a similarly small, non-significant effect (*d* = 0.15); interestingly, however, the effect was descriptively in the opposite direction compared to the earlier findings (Ebert et al., 2024a). Hence, the current findings call into question the robustness of the effect observed by Ebert et al. (2024a), and they cast doubt on our earlier conclusion that kindergarten children hold implicit gender stereotypes about spatial ability at preschool age. There are several plausible explanations for the divergent findings across the two studies. One possibility is that they reflect cohort effects, potentially linked to shifts in public discourse and evolving societal values. For example, Block et al. (2022) demonstrated that even brief exposure to stereotypical or counter-stereotypical narratives can influence implicit gender stereotypes in children aged six to eleven. This suggests that increased awareness and discussion of gender stereotypes among parents, educators, or in media may shape children’s implicit associations—possibly even within short timeframes. Finally, it is worth emphasizing that the effect sizes observed in both studies were very small, raising the possibility that young children may not hold stable or robust implicit stereotypes about spatial ability at this age.

### 4.2 Visuospatial working memory

We found significant correlations between our measure of visuospatial working memory (VSWM), mental rotation (MR) accuracy, and IAT scores. This aligns with previous research showing a robust relationship between VSWM and MR performance in both children (Lehmann et al., 2014) and adults (Kaufman, 2007; Hofmann et al., 2024). Notably, these studies have specifically linked Corsi span measures to MR outcomes, reinforcing the idea that individual differences in spatial working memory capacity contribute to spatial reasoning abilities.

### 4.3 The relation of gender stereotypes and mental rotation performance

Our results show no relation of gender stereotypes about spatial ability and MR response times. This aligns with findings from our previous study (Ebert et al., 2024a), in which we also observed no effects of either implicit or explicit gender stereotypes on MR response times in a mixed-gender sample. Also, akin to results from the same study, we did find an effect of implicit gender stereotypes on MR accuracy in the current sample. Interestingly, the direction of the effect was reversed: girls who more strongly associated spatial ability with boys performed more accurately than those who associated it with their own gender. Accordingly, we reject our main hypotheses regarding the relationship between implicit gender stereotypes and MR performance. This unexpected direction of this effect is difficult to reconcile with existing theoretical frameworks (e.g., Schmader, 2010). However, it is plausible that stereotypes are in development in the researched age group, or that they are not applied to the self (Ebert et al., 2024a) and therefore do not impact performance in commonly observed ways.

### 4.4 Limitations

It is important to note that the MR tasks used in this study was not identical to the task in Ebert et al. (2024a), where children were asked to decide whether two drawings were identical or mirror images of each other. In contrast, the current study required children to match a target drawing to one of two reference images. This adaptation was intended to increase comprehensibility for young children and to yield more analyzable data, as it eliminated the need to discard mirror-image trials. However, there was some indication of a potential ceiling effect in MR accuracy in the present study, suggesting that the modified task may have been too easy. Nonetheless, model assumptions were carefully examined, and no evidence of substantial model misspecification was found. It should also be noted that the target sample size was not met, limiting our ability to reliably detect effects of interest. The used IAT rests on the assumption that an association between spatial toys (representative of spatial ability) and gender is measured, an assumption that cannot be confirmed within the confines of this study. Combining implicit and explicit measures in future research may in part alleviate this issue.

## 5 Conclusion

Taken together, our findings call into question, whether implicit gender stereotypes about spatial ability are reliably present in preschool-aged kindergarten girls. Moreover, they do not support the hypothesis that such stereotypes are meaningfully related to spatial task performance. We could not replicate a previously observed effect of implicit stereotypes on MR accuracy. In fact, the direction of the effect was reversed in the present study, complicating interpretation. Given the small and inconsistent effects across studies, and the methodological differences between them, we urge caution in drawing firm conclusions about the presence or impact of such stereotypes at this early developmental stage. The results do, however, suggest that kindergarten years may hold potential in shaping children’s resilience against stereotypes. If stereotypes are not fully developed at this stage, parents and caretakers may successfully intervene in structured or unstructured ways to foster un-stereotyped thinking. Future research should seek to clarify under what conditions, if any, implicit gender stereotypes influence spatial cognition in young children. This may include larger samples, alternative measures of implicit bias ideally, combined with explicit measures (e.g., structured interview or questionnaire), and longitudinal approaches that capture developmental changes over time. Future studies should also aim for measures of MR with adequate difficulty to avoid floor- and ceiling effects. For instance, the difficulty of the task used in this study could be adapted through the inclusion of more rotation angles. Pilot testing or comparative studies probing different MR tasks to identify sensible difficulty levels could prove helpful. Moreover, investigating the role of parents’, teachers’ or caretakers’ stereotypes could provide further insight into the complex relationships between stereotyping and children’s spatial ability.

## Data Availability

The raw data supporting the conclusions of this article will be made available by the authors, without undue reservation.
