# Supplementary material for: No relationship between gender stereotypes and mental rotation in preschool girls
Source: Front Psychol. 2025 Sep 16;16:1650979. doi: 10.3389/fpsyg.2025.1650979 (PMC12481608; doi:10.3389/fpsyg.2025.1650979)
Supplement: Supplementary file 1 [file Table_1.docx]

**Supplementary Material**

**Figure S1**

*Frequency of the Proportion of Correct Responses on the Mental Rotation Task*
