# Supplementary figures and images for: No relationship between gender stereotypes and mental rotation in preschool girls
Source: Front Psychol. 2025 Sep 16;16:1650979. doi: 10.3389/fpsyg.2025.1650979 (PMC12481608; doi:10.3389/fpsyg.2025.1650979)

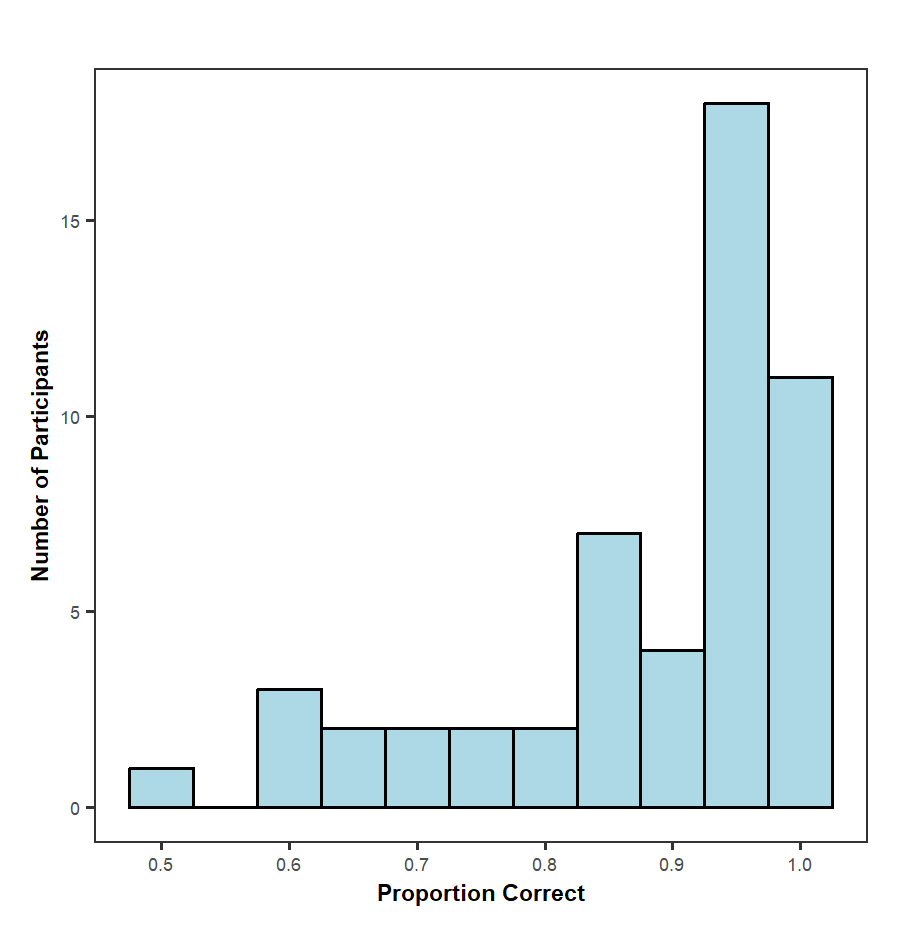

Supplement: Supplementary file 2 [file Image_1.tif]
